# Temperature and predators as interactive drivers of community properties

**DOI:** 10.1002/ece3.10665

**Published:** 2023-10-31

**Authors:** John P. DeLong, Kyle E. Coblentz, Stella F. Uiterwaal, Chika Akwani, Miranda E. Salsbery

**Affiliations:** ^1^ School of Biological Sciences University of Nebraska – Lincoln Lincoln Nebraska USA; ^2^ Present address: Living Earth Collaborative Washington University in St. Louis St. Louis Missouri USA; ^3^ Present address: Rochester Institute of Technology K‐12 University Center Rochester New York USA

**Keywords:** biodiversity ecosystem function, biomass pyramid, ecosystem function, temperature–size rule, thermal performance, trophic cascades, warming

## Abstract

The effects of warming on ecological communities emerge from a range of potentially asymmetric impacts on individual physiology and development. Understanding these responses, however, is limited by our ability to connect mechanisms or emergent patterns across the many processes that drive variation in demography. Further complicating this understanding is the gain or loss of predators to many communities, which may interact with changes in temperature to drive community change. Here we conducted a factorial warming and predation experiment to test generalized predictions about responses to warming. We used microcosms with a range of protists, rotifers, and a gastrotrich, with and without the predator *Actinosphaerium*, to assess changes in diversity, body size, function, and composition in response to warming. We find that community respiration and predator:prey biovolume ratios peak at intermediate temperatures, while species richness declined with temperature. We also found that overall biomass increased with species richness, driven by the effect of temperature on richness. There was little evidence of an interaction between predation and temperature change, likely because the predator was mostly limited to the intermediate temperatures. Overall, our results suggest that general predictions about community change are still challenging to make but may benefit by considering multiple dimensions of community patterns in an integrated way.

## Graphical abstract

The effects of warming on ecological communities emerge from a range of potentially asymmetric impacts on individual physiology and development. We used microcosms with a range of protists, rotifers, and a gastrotrich, with and without the predator Actinosphaerium, to assess changes in diversity, body size, function, and composition in response to warming. We find that community respiration and predator:prey biovolume ratios peak at intermediate temperatures, while species richness declined with temperature. We also found that overall biomass increased with species richness, driven by the effect of temperature on richness.

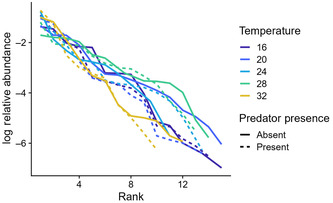

## 1 INTRODUCTION

Increasing temperatures can alter ecological systems through direct effects on organism physiology (Brown et al., 2004), developmental effects on phenotypes (Atkinson, 1994), or indirect effects on species interactions (Englund et al., 2011). The vast array of potentially asymmetric effects of temperature on traits and interactions may lead to unpredictable and broad impacts of warming on complex ecological systems (Damien & Tougeron, 2019; Dell et al., 2014; Gibert et al., 2022; McLean et al., 2016; Mordecai et al., 2019; Shurin et al., 2012). Some predictions, however, do emerge from dynamic consumer‐resource, food web, or competition models depending on the emergence of thermal effects from individuals to species interactions to community processes and structure.

Concurrent with warming are species invasions, predator losses, and range shifts throughout the world, generating novel communities and species interactions in many places (Croll et al., 2005; Dorcas et al., 2012; Elmhagen et al., 2015; Montserrat et al., 2013). Species interactions, including predator–prey interactions, can be strongly temperature dependent (Burnside et al., 2014; Rall et al., 2012; Uiterwaal & DeLong, 2020), which means that changes in temperature and predation are likely to interact in the way they shape communities (Beveridge et al., 2010; Rasher et al., 2020; Ross et al., 2022). Furthermore, thermal limits may vary across trophic levels, suggesting another potential way in which warming may interact with predation to reshape communities (da Silva et al., 2023). There is thus a growing need to understand how predation interacts with warming to modify ecological communities. Here we conducted a factorial experiment to assess the potential interactions between predation and temperature on a set of integrated community properties.

### 1.1 Why should community properties respond in an integrated way to temperature?

**FIGURE 1:**
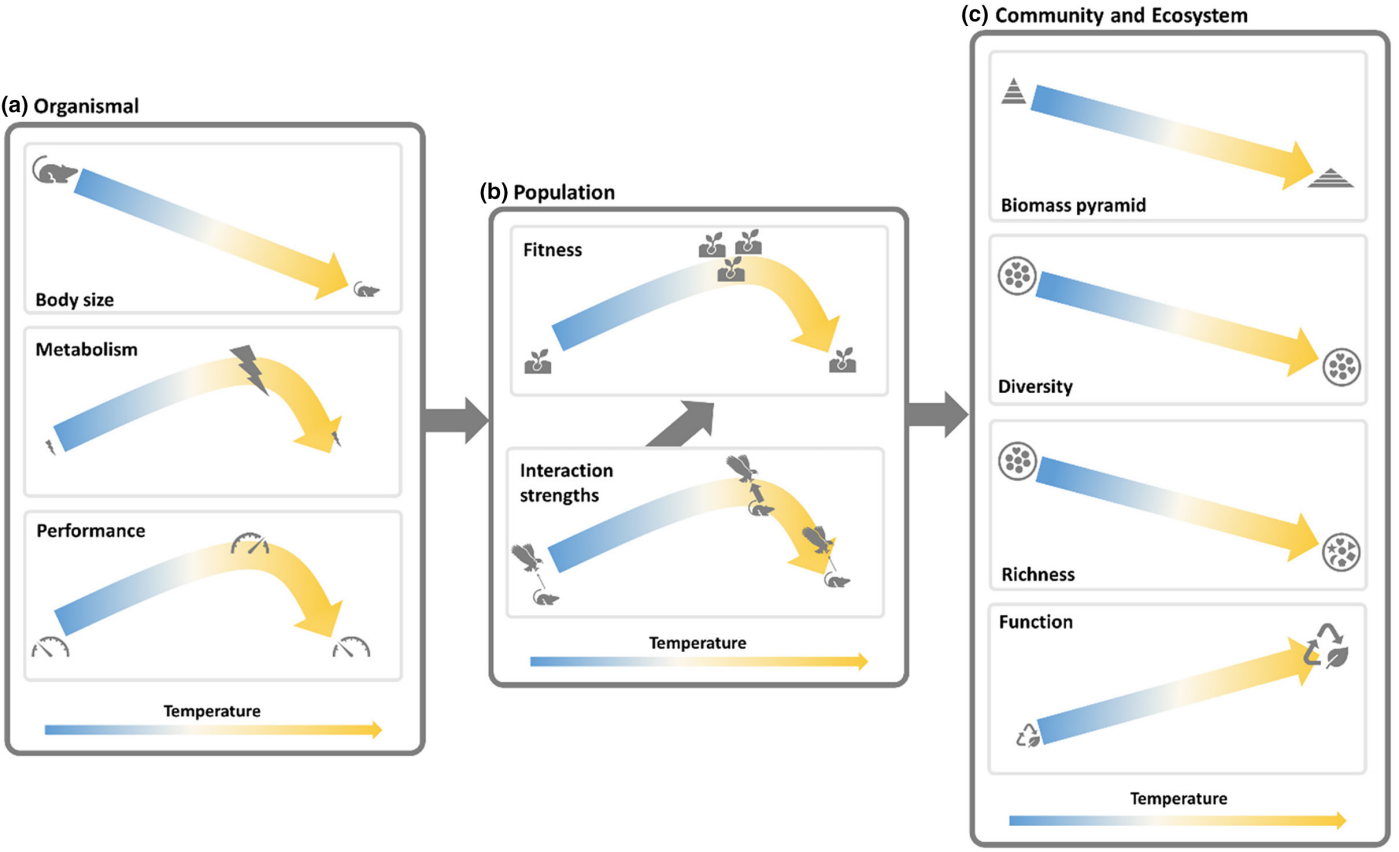
Schematic of some expected effects of temperature on organisms (a), population (b), and community and ecosystems (c). Effects occurring at the organismal level combine to influence population‐level patterns, which combine to influence community‐ and ecosystem‐level patterns.

There are several key individual‐level effects of warming that may have consequences for community structure. For example, body size of a wide range of ectotherms declines with temperature during development (Figure 1), a pattern known as the temperature–size rule (TSR; Atkinson, 1994; DeLong, 2012; Sheridan & Bickford, 2011). Despite some claims to universality of the TSR, there are many exceptions that may complicate how temperature‐influenced body size alters community processes (Atkinson, 1995; DeLong et al., 2017; Gardner et al., 2011). Another major expectation is of an increase in metabolic rate with warming (Brown et al., 2004). This increase, in most cases, is likely to peak and begin to decline again, following a pattern referred to as a thermal performance curve, where the peak itself often occurs within the biologically relevant temperature range for the organism (DeLong et al., 2018; Figure 1). Temperature also may influence mortality rates (McCoy & Gillooly, 2008), potentially limiting species to certain environments or just altering abundances if the mortality is not offset by increases in reproduction.

Organismal level responses to temperature have population‐level consequences, and the most common expectations for these are that both population growth rate and interaction strengths peak at intermediate temperatures (Figure 1). As with metabolic rate, the typical relationship between population growth rate and temperature is unimodal (Angilletta, 2009; Huey & Berrigan, 2001; Ratkowsky et al., 2005). This pattern emerges directly from the effects of temperature on life history and the resulting changes in birth and death rates (Luhring et al., 2018). Lower demographic performance of most organisms at higher temperatures implies a sensitivity to thermal extremes very broadly, indicating that climate change is likely to reduce species diversity wherever temperatures range too high (Kingsolver et al., 2013; Vasseur et al., 2014). Both the initial rise and the asymptote of the functional response (relationship between foraging rate and prey density) are also nonlinearly related to warming, generally leading to peak interaction strengths at intermediate temperatures (Englund et al., 2011; Uiterwaal & DeLong, 2020; Uszko et al., 2017). Such temperature dependence of the functional response can have strong impacts on the dynamics and overall abundance of predators and their prey (DeLong & Lyon, 2020; Vasseur & McCann, 2005).

Population‐level processes generate emergent outcomes at the community and ecosystem scales (Beveridge et al., 2010; Garzke et al., 2019; Gebert et al., 2022; Figure 1). Warming is thought to shuffle the composition of communities as species' upper critical temperatures are passed, resulting in species losses, potentially with negative impacts on ecosystem services (Chapin III et al., 2000; Kingsolver et al., 2013; Malcolm et al., 2006). However, across elevational and latitudinal gradients, we see increased diversity at warmer temperatures (Peters et al., 2016) or unimodal responses of species richness to temperature (Rahbek, 1995), suggesting that warming may not universally lead to lower species diversity. Because biodiversity promotes ecosystem function, warming is also expected to affect ecosystem function via its effects on community composition or species traits (Gebert et al., 2022). Thus, species losses associated with warming are often linked to decreases in function (García et al., 2018; van der Plas, 2019). Conversely, some metrics of ecosystem function are expected to increase with warming, including respiration at the community level (Jankowski et al., 2014; Smith et al., 2019; Yvon‐Durocher et al., 2012). Asymmetric responses of populations at different trophic levels, in part possibly an outcome of the temperature dependence of interaction strengths, may lead to changes in biomass pyramids, with predictions typically suggesting top‐heavy pyramids (i.e., more biomass at higher trophic levels) at warmer temperatures or top‐heavy pyramids at intermediate temperatures (Bideault et al., 2021; Shurin et al., 2012). Warming experiments and studies of natural systems confirm some of these predictions, but few experiments have addressed the broad set of changes in ecological communities in response to warming (Kratina et al., 2012; O'Connor et al., 2009; Petchey et al., 1999; Shurin et al., 2012; Yvon‐Durocher et al., 2011). Critically, since a common set of individual‐ and population‐level responses underlie community‐level responses, many community‐level processes may be intrinsically (mechanistically) linked, even feeding back to individual‐level properties (Daufresne et al., 2009; Thakur et al., 2018), and therefore understanding these responses may require an integrative approach, or barring true integration, at least one which views a collection of responses together.

Here we report on a factorial microcosm experiment across a temperature gradient with and without predators present. Because the communities consist of small organisms with generation times on the order of hours and days, populations were free to grow or decline and the predators responded at both the individual and population levels. Thus, predator populations were both a treatment (presence/absence) and a response (abundance, cell volume, and biovolume). We then assessed the myriad changes caused by temperature for both predator and no‐predator dishes and evaluated a collection of predictions and expectations of community change gleaned from the literature. Specifically, we tested five generalized predictions for the impacts of warming: that increased temperature will (1) reduce species diversity, (2) reduce average body sizes of individuals within species and cause a shift toward smaller species in the community, (3) alter community composition, (4) lead to more top‐heavy biomass pyramids, here measured as the ratio of predator biovolume to prey biovolume, and (5) increase the total system respiration. We also assess whether the presence or absence of a generalist predator alters any of the observed patterns and whether predator effects are themselves temperature‐dependent. We find broad effects of temperature only partly in line with expectations, and that predators have their largest effects at the temperature where they do best. Otherwise, predator effects interacted very little with temperature effects, but we suspect this was due to the particular identity and foraging strategy of the predator.

## 2 METHODS

### 2.1 Experiments

We collected ciliates, rotifers, and a gastrotrich from various ponds near Lincoln, Nebraska, United States, during the summer of 2021. Ponds were generally about one hectare or smaller with emergent vegetation and no active watercraft use. We cultured species in media made with Protozoan pellets (Carolina Biological Supply) dissolved in filtered and autoclaved pond water from the collection sites. The media were inoculated with a range of bacterial species collected from the original sites and plated onto a standard agar plate by filtering a mix of pond water through a 5‐μm syringe filter onto the plate. We also added 0.7 g of dried, autoclaved, and ground pond mud to increase the availability of rare minerals and nutrients. Of the species we collected, 16 morphospecies (14 ciliates and two rotifers) grew sufficiently well to include in the experiment; the gastrotrich appeared unexpectedly during the experiment. The ciliates were *Paramecium caudatum*, *P. aurelia*, *P. bursaria*, *Halteria* sp., *Frontonia* sp., *Euplotes* sp., *Coleps hirtus*, *Colpidium* sp., *Colpoda* sp., *Stylonichia* sp., *Urostyla* sp., *Vorticella* sp., and two unidentified morphospecies. The rotifers were a bdelloid species and a *Euchlanis* sp. The species of gastrotrich was unknown, but as detritivores that hatch from unfertilized eggs live only a few days, and can be consumed by *Actinosphaerium*, we considered them to be ecologically complementary to the ciliates in the experiment. We initially combined groups of species to determine which would persist in communities, and then combined these different communities into one larger community “stock” with which to initiate the experimental units. We purchased the predator, *Actinosphaerium* sp. (hereafter just *Actinosphaerium*), from Carolina Biological Supply and maintained them in the same media with a variety of smaller ciliates and rotifers added as prey. We have found *Actinosphaerium* locally at these same collection sites but did not have locally‐collected stocks available at the start of the experiment.

We implemented a factorial combination of five temperatures (16, 20, 24, 28, and 32°C) and predator/no‐predator treatments, replicated three times, for 30 total experimental microcosms. The microcosms were 50‐mm diameter Petri dishes containing 8 mL of the community stock with an additional 0.3 mL of water from the predator stock. For predator dishes, we added five individual *Actinosphaerium* cells in the 0.3 mL of water, and for no‐predator dishes, we added the 0.3 mL of media without cells to ensure that prokaryotic species from the predator stock were present in all dishes. In two of the no‐predator dishes, however, *Actinosphaerium* cells were inadvertently added, likely as cysts, converting two microcosms at 24°C to the predator treatment.

We marked the water level in the dishes with a permanent marker to have a reference point for controlling microcosm volume (the total 8.3 mL). If evaporative water loss brought the water level below the mark, we topped off the dishes with autoclaved pond water prior to sampling. Because we maintained the relative humidity at 70%, evaporative water loss was minimal. We sampled all dishes three times per week (Monday, Wednesday, and Friday) throughout the experiment for 15 days. We were less interested in the dynamics and more in the ultimate steady‐state conditions within the microcosms. We therefore tracked abundances in the predator dishes to determine when the system had settled and when to do larger scale destructive sampling. Although we sampled all dishes, we counted cells only in the predator dishes through the first 12 days due to time constraints. At each sampling, we estimated the abundance of each morphospecies either through a complete census of the dish, if the species was relatively rare, or through a count in a 0.1 mL of well‐mixed sample, if the species was relatively abundant. We pulled an additional 0.4 mL each day and replaced the removed 0.5 mL total with 0.5 mL of new, bacterized protozoan media. We took separate 0.1 and 0.4 mL samples because 0.1 mL was sufficient for subsample counting, but we used the full 0.5 mL volume for media turnover and replacement. On Days 13 and 15, we counted all predator and no‐predator dishes, and did extra counts of *Frontonia* sp. on Day 14.

At the end of the experiment, we performed two types of destructive sampling. First, we measured the rate of oxygen consumption for each sample at its experimental temperature using a PreSens (Germany) SDR respirometer. To do this, we pipetted 2.5 mL of culture media (~1/3 of each culture) into a 2.87‐mL SensorVial, filling the rest with autoclaved pond water to ensure no air bubbles were present in the vial. We placed all vials for a given temperature and a control vial with only autoclaved pond water into a SensorDish tray. We placed the tray into a temperature‐controlled chamber at the appropriate experimental temperature and measured oxygen concentrations in the vials for approximately 1 h in the dark. To account for any system equilibration, we only used oxygen data collected from minutes 30 to 55, during which time we took measurements every 2 min. For each vial, we obtained a slope of oxygen use over time using ordinary least squares regression in Matlab 2022b, subtracting the slope of the control vial to account for any background changes in oxygen. Second, we measured cell volumes of the remaining community members in the microcosms after pulling samples for respiration using a FlowCam for ciliates, rotifers, and gastrotrichs and photographs taken using a Leica M165C stereo microscope with a Leica DMC4500 camera for *Actinosphaerium*. We calculated ciliate cell, rotifer, or gastrotrich volume as the equivalent spherical diameter (ESD) using the FlowCam software. We calculated *Actinosphaerium* biovolumes using their widths assuming that they are spherical. We calculated morphospecies‐level biovolumes as the product of average abundance and average individual biovolume and community‐level biovolume as the sum of biovolume for all present species.

### 2.2 Analysis

#### 2.2.1 Body size, diversity, biomass pyramids, and respiration

We averaged the abundance data during Days 13–15 to assess the effect of our treatments on the community. We used generalized additive models (GAMs) to evaluate our hypotheses on the effects of temperature and predation on diversity, body sizes, biovolume pyramids, and system respiration. We used GAMs because we a priori expected nonlinear relationships between variables but did not know exactly what the shapes of these relationships might be. We fit GAMs in a model selection framework to assess the joint effects of temperature, the presence or absence of predator, and their interaction on the biovolumes of morphospecies and the community‐ and ecosystem‐level responses. In particular, for each response variable considered except biovolume ratio, we fit five separate models: (1) a model with a smooth effect of temperature, an effect of predator presence, and an interaction between temperature and predator presence, (2) a model with a smooth effect of temperature and an effect of predator presence with no interaction, (3) a model with only a smooth effect of temperature, (4) a model with only an effect of predator presence, and (5) a “null” intercept‐only model. Because biovolume ratios could only be calculated with the predator present, when biovolume ratio was the response variable we only fit a model with a smooth effect of temperature and a “null” intercept‐only model. For the morphospecies biovolume analyses, the average biovolume for a morphospecies within a particular treatment had variable sample sizes used to calculate the average depending on the number of organisms within the FlowCam sample. Therefore, in the GAMs, we weighted each average biovolume by the number of organisms used to calculate the average. We also excluded morphospecies with size observations in fewer than eight of the replicates and one morphospecies, *Euplotes*, because its shape (flat and rectangular) led to unreliable volume estimates from the FlowCam. To fit each of the GAMs to the data, we used the R package “mgcv” (Wood, 2017). Because temperature took a maximum of six different values in our experiment, we set the potential upper limit of the degrees of freedom for the temperature smooth in the GAMs to the number of temperature values minus one (note that some morphospecies only had biovolumes for a subset of the temperatures considered in the overall experiment). We used AICc to perform model comparison due to a generally low ratio of sample sizes to potential parameters.

#### 2.2.2 Population abundance

To evaluate how predators and temperatures influenced population abundance across species, we conducted a general linear model on the log of frequencies against rank. We fitted a model with predator presence/absence and temperature as factors, and with both predictors affecting the slope of the response as an interaction between rank and factor, using the ‘lm’ function in R.

#### 2.2.3 Community composition

To evaluate how temperature and predation altered prey community composition, we used ordination and statistical tests on the associated distance matrices. To visualize how community composition changed with temperature and predation, we used nonmetric multidimensional scaling (NMDS) with a distance matrix calculated using Bray–Curtis dissimilarity of the Wisconsin double standardized square root transformed abundance data of the morphospecies. We used partial Mantel tests to examine whether changes in community composition were associated with temperature or predation. Partial Mantel tests examine the correlation between two distance matrices while accounting for the correlations with a third distance matrix. In our case, we examined the correlation between the community composition distance matrix used for the ordination and a distance matrix of Euclidean distances between temperatures accounting for a distance matrix of Euclidean distances between *Actinosphaerium* abundances and vice versa.

We performed the ordination using the “vegan” package and the partial Mantel tests using the “phytools” package in R (Oksanen et al., 2022; Revell, 2012). All other analyses were performed using R (v. 4.2.2). The data and code for the analyses are available as a permanently archived GitHub repository on Zenodo will be made available upon acceptance as a permanently archived GitHub repository on Zenodo but are included as a supplementary zipped folder during the review process.

## 3 RESULTS

### 3.1 Body size

**FIGURE 2:**
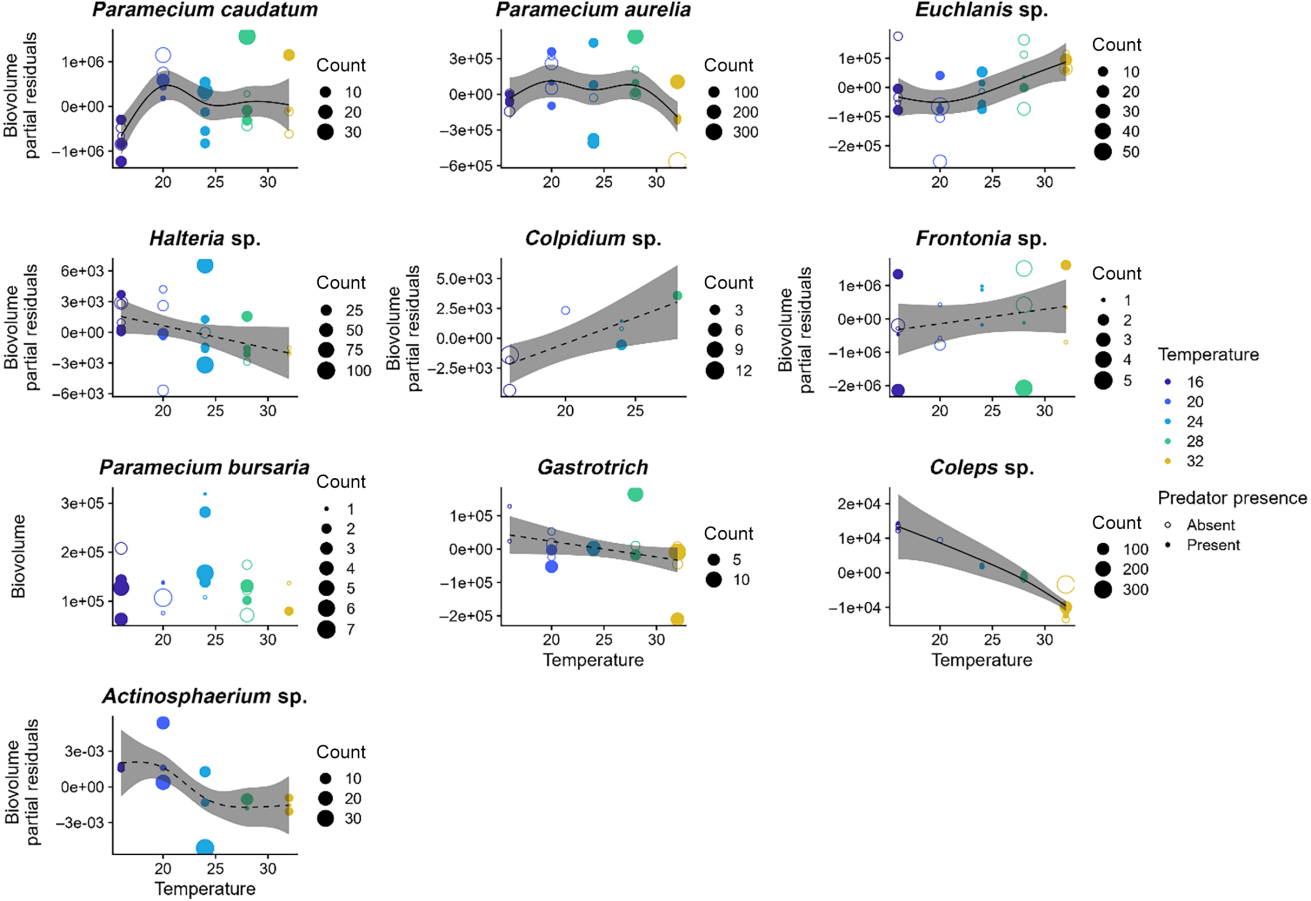
Species showed mixed cell biovolume responses to temperature. Some species (e.g., *Euplotes* sp. and *Coleps hirtus*) showed decreases in cell biovolumes, whereas others (*Paramecium caudatum* and *P. aurelia*) showed unimodal responses, and others (e.g., *Euchlanis* sp.) showed biovolume increases. Solid lines give the fitted response of biovolumes to temperature for models where the temperature‐only model had a ΔAICc value >2 from the next closest model while dashed lines give the fitted response where the temperature‐only model and the null model were within two ΔAICc units. The shaded areas correspond to 95% confidence intervals.

Overall, our FlowCam and photograph analysis generated enough biovolume estimates to analyze temperature–size relationships for 9 of the 17 morphospecies found in the communities and the predator, *Actinosphaerium*. Individual biovolume estimates ranged from 30 for *Colpidium* to 723 for *Paramecium aurelia*, and the number of replicates with biovolume estimates ranged from 8 for *Colpidium* to all 30 for *Paramecium caudatum*. In general, we found no consistent support for the temperature–size rule that body size (here biovolume) should decrease with increasing temperatures (Figure 2, Table 1). Rather, some morphospecies showed declines in biovolume with temperature (specifically *Halteria* sp., the gastrotrich, *Coleps hirtus*, and *Actinosphaerium*) whereas all others showed increases, unimodal relationships, or no relationship. We also found no evidence of a general effect of predation or its interaction with temperature on biovolumes (Figure 2, Table 1). Of the nine prey morphospecies with adequate sample sizes to examine the relationship between temperature, predation, and biovolume, one of the nine (*Paramecium bursaria*) showed no clear support for the temperature model relative to the null, intercept‐only model (Table 1). For four of the nine morphospecies (*Halteria*, gastrotrichs, *Colpidium*, and *Frontonia*), the temperature‐only model and the null model were within two ΔAICc units (Table 1). Of these four morphospecies, *Halteria* and gastrotrichs showed trends toward a decrease in biovolume with increasing temperatures while *Colpidium* and *Frontonia* showed a trend toward increasing biovolume with increasing temperatures (Figure 2). The remaining four species (*Paramecium caudatum*, *Paramecium aurelia*, *Euchlanis*, and *Colpoda*) showed clear support for the model including only temperature (Table 1). These morphospecies showed variable volume–temperature relationships (Figure 2). In particular, *Paramecium caudatum* and *Paramecium aurelia* showed nonlinear, unimodal biovolume–temperature relationships, *Euchlanis* showed an increasing biovolume–temperature relationship, and *Colpoda* showed a decreasing biovolume–temperature relationship (Figure 2). For the predator, *Actinosphaerium*, the temperature‐only model and the null model were within two ΔAICc units and the relationship between biovolume and temperature showed a trend toward a nonlinear, decreasing biovolume–temperature relationship (Figure 2, Table 1).

**TABLE 1:** AICc and ΔAICc for the models of biovolume responses to temperature and predator presence for each species.

Model	AICc	ΔAICc
*Paramecium caudatum*		
Temperature	895.1	0
Temperature, predation, and interaction	896.1	1.0
Temperature, predation, no interaction	898.3	3.2
Null	902.2	7.1
Predation	904.6	9.5
*Paramecium aurelia*		
Temperature	832.7	0
Temperature, predation, no interaction	834.9	2.2
Null	836.9	4.2
Predation	838.8	6.1
Temperature, predation, and interaction	838.9	6.2
*Euchlanis* sp.		
Temperature	707.6	0
Temperature, predation, and interaction	709.7	2.1
Temperature, predation, and no interaction	710.6	3.0
Null	712.5	4.9
Predation	715.0	7.4
*Halteria* sp.		
Temperature	497.2	0
Null	498.2	1.0
Temperature, predation, and no interaction	500.0	2.8
Predation	500.4	3.2
Temperature, predation, and interaction	501.7	4.5
*Colpidium* sp.		
Temperature	156.2	0
Null	157.1	0.9
Predation	160.0	3.8
Temperature, predation, and no interaction	163.9	7.7
Temperature, predation, and interaction	226.6	70.4
*Frontonia* sp.		
Null	556.0	0
Temperature	557.7	1.7
Predation	558.9	2.9
Temperature, predation, and no interaction	561.1	5.1
Temperature, predation, and Interaction	565.0	9.0
*Paramecium bursaria*		
Null	527.2	0
Predation	529.3	2.1
Temperature	531.1	3.9
Temperature, predation, and no interaction	533.8	6.6
Temperature, predation, and interaction	537.3	10.1
*Gastrotrich*		
Temperature	608.8	0
Null	609.6	0.8
Temperature, predation, and no interaction	611.1	2.3
Predation	611.4	2.4
Temperature, predation, and Interaction	616.0	7.2
*Coleps hirtus*		
Temperature	393.1	0
Temperature, predation, and No interaction	396.5	3.4
Temperature, predation, and interaction	398.8	5.7
Null	409.3	16.2
Predation	412.1	19.0
*Actinosphaerium* sp.		
Null	−120.5	0
Temperature	−120.3	0.2

### 3.2 Population abundance

**FIGURE 3:**
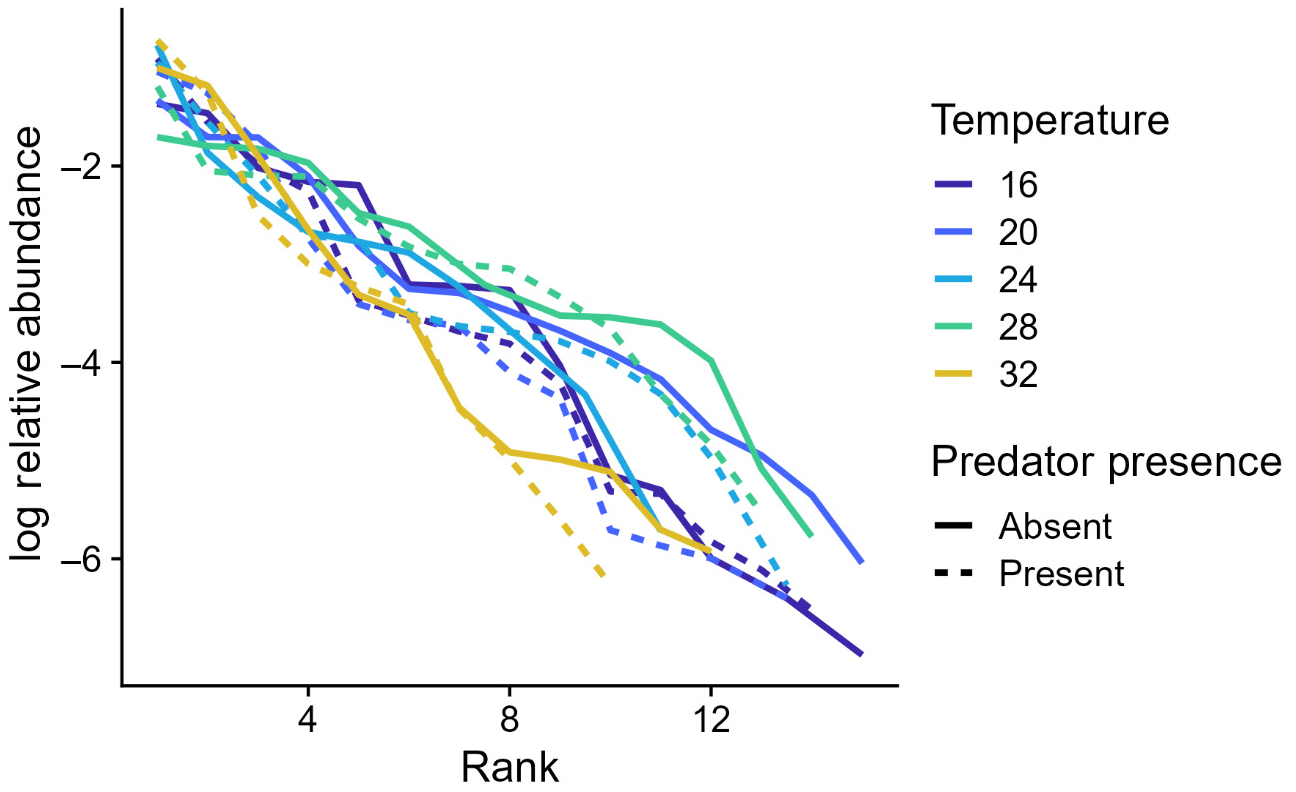
Population relative abundance responses to temperature and the presence of predators. Rank abundance curves for predator/no predator dishes and dishes at all five temperatures were nearly completely overlapping, but with some differences across temperatures.

**FIGURE 4:**
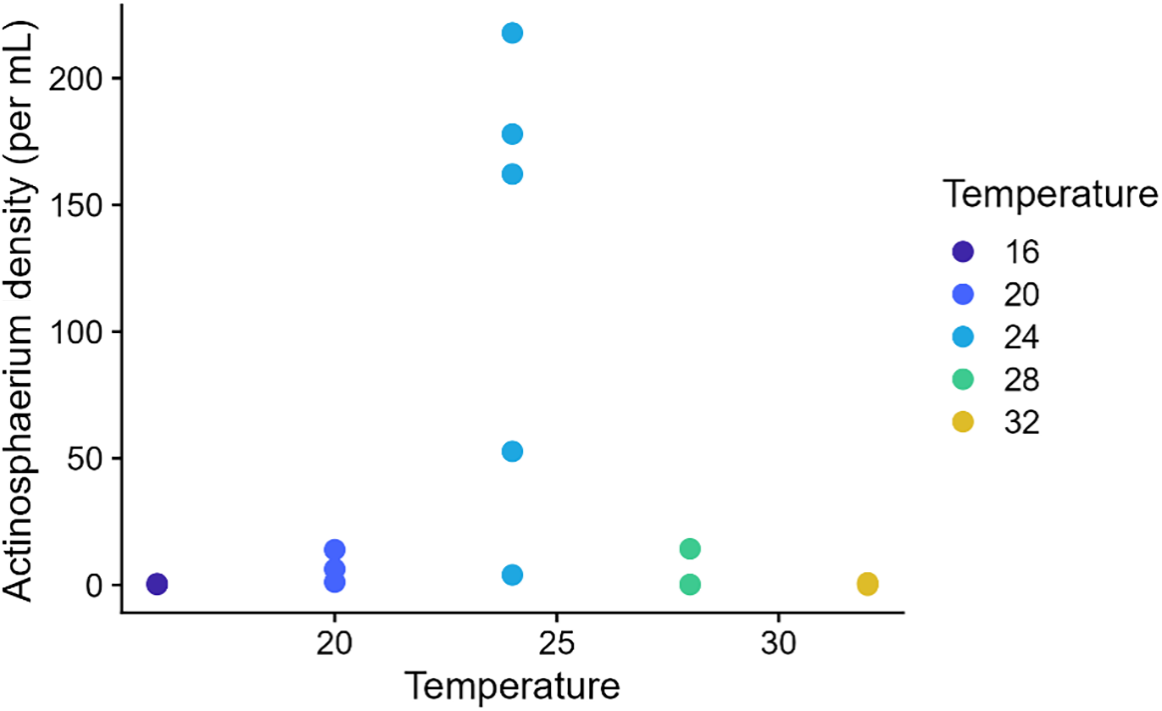
The abundance of the predator *Actinosphaerium* at different temperatures. The predators went extinct at the hottest and coldest temperatures.

The abundance of species in the microcosms showed typical hollow rank abundance curves (McGill et al., 2007). Although very similar, rank abundance curves in the predator dishes fell off more quickly than those in the no predator dishes (predation: rank interaction term: *t* = −3.740, *p* = .0002; Figure 3). Likewise, rank abundance curves were broadly overlapping across temperatures but still showed temperature‐specific slopes. Relative to 16°C, curves at 20°C (temperature: rank interaction term: *t* = 3.35, *p* < .001) and 28°C (*t* = 5.88, *p* < .001) had shallower curves, and curve at 32°C had a steeper curve (*t* = −3.740, *p* = .0002; Figure 3). For the predator, *Actinosphaerium*, population abundances peaked at intermediate temperatures, and the predator was lost from all dishes at the coldest and hottest temperatures (Figure 4).

### 3.3 Diversity

**FIGURE 5:**
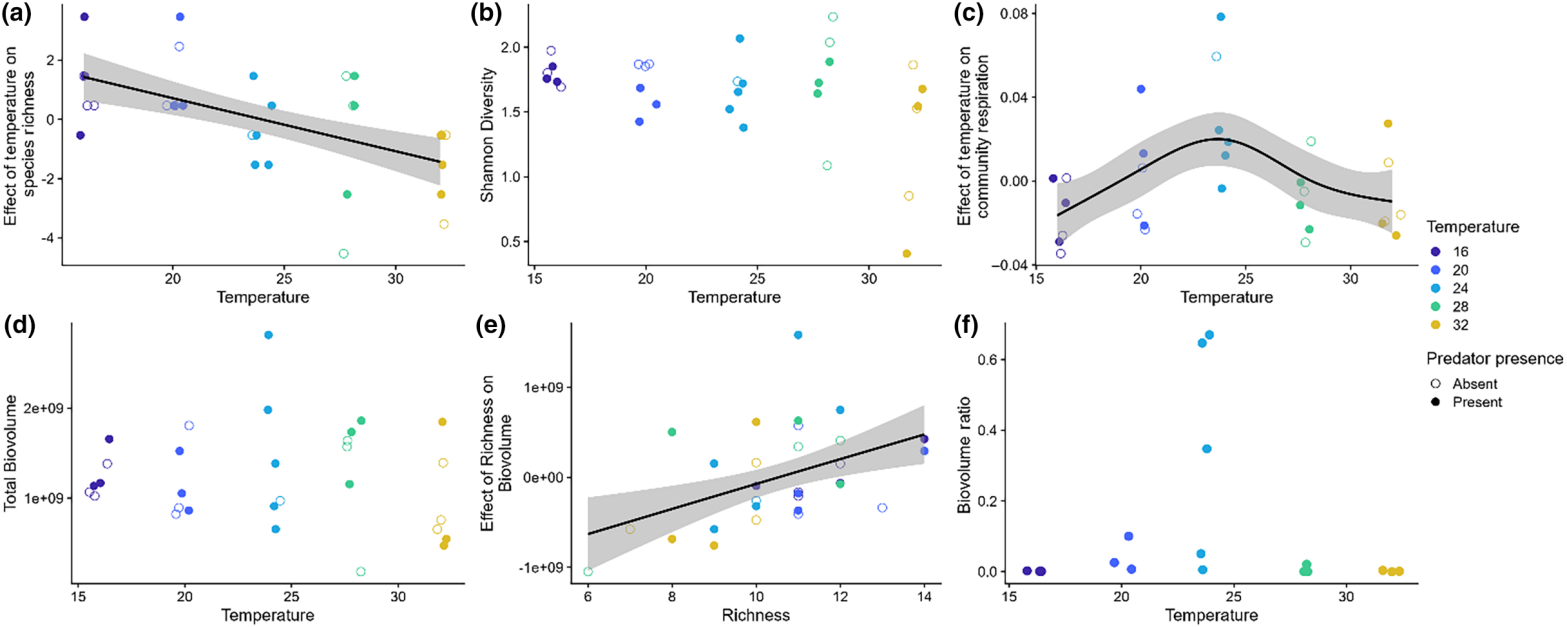
Community and ecosystem responses to temperature and the presence of predators. Species richness declined with temperature (a), but Shannon diversity was unaffected by temperature (b). Community‐level respiration peaked at the intermediate temperature (c) even though biovolume was independent of temperature (d). Total community‐level biovolume increased with species richness (e) but the ratio of predator biovolume to prey biovolume only suggested a peak at intermediate temperatures (f).

Species richness decreased with temperature with no statistical support for an effect of predation or its interaction with temperature (Figure 5a, Table 2). In contrast, we found no support for effects of temperature or predation on Shannon diversity relative to a null, intercept‐only model (Figure 5b, Table 2).

**TABLE 2:** AICc and ΔAICc for the community and ecosystem responses.

Model	AICc	ΔAICc
Temperature and species richness		
Temperature	116.0	0
Temperature, predation, no interaction	118.5	2.5
Temperature, predation, and interaction	121.3	5.3
Null	124.9	8.9
Predation	127.3	9.3
Temperature and Shannon diversity		
Temperature	26.4	0
Null	27.4	1.0
Temperature, predation, no interaction	28.3	1.9
Predation	29.0	2.6
Temperature, predation, and interaction	31.3	4.9
Temperature and respiration		
Temperature	−130.1	0
Null	−127.8	2.3
Temperature, predation, no interaction	−127.0	3.1
Predation	−126.3	3.8
Temperature, predation, and interaction	−119.6	10.5
Temperature and total biovolume		
Null	1296.2	0
Predation	1297.1	0.9
Temperature	1299.1	2.9
Temperature, predation, no interaction	1299.8	3.6
Temperature, predation, and interaction	1303.3	7.1
Richness and total biovolume		
Richness	1291.5	0
Null	1296.2	4.7
Temperature and biovolume ratio		
Null	1.0	0
Temperature	3.9	2.9

### 3.4 Community structure

**FIGURE 6:**
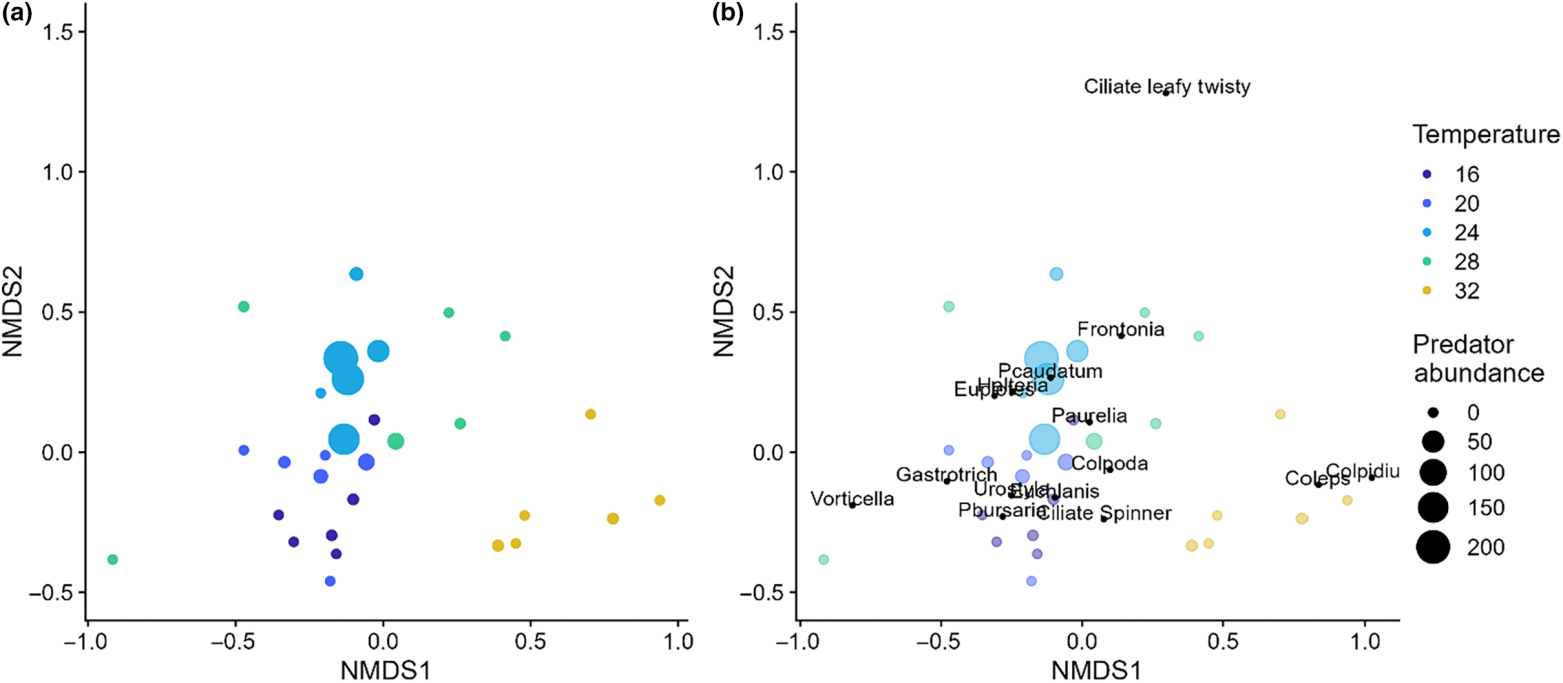
Multivariate depiction of community composition with nonmetric multidimensional scaling (NMDS). Both plots show the same data and axes, with (a) not showing species names for clarity and (b) showing species names to identify their locations.

We found evidence for a correlation between community composition distances and temperature distances when accounting for predator density distances (partial Mantel test; *p* = .001) but not for a correlation between community composition distances and predator density distances when accounting for temperature distances (partial Mantel test; *p* = .48). The ordination also revealed species whose relative abundances within communities appeared responsive to temperature (Figure 5). For example, *Vorticella*, Bdelloid rotifers, and *Urostyla* had greater relative abundance contributions near colder temperature samples in the ordination, whereas *Coleps*, *Colpidium*, and *Stylonichia* had greater relative abundance contributions near the warmest temperature samples (Figure 6b). Despite the lack of a significant correlation between community composition distances and predator density distances, several of the species with greater relative abundance contributions near the communities with high predator densities are those that are resistant to predation by *Actinosphaerium* and for which we did not observe any predation events. These species are resistant either because they are very small and have an effective escape mechanism (e.g., *Halteria* jumping) or because they are too large and/or too strong of swimmers for *Actinosphaerium* to capture (e.g., *Frontonia* and *Paramecium caudatum*).

### 3.5 Biovolume

We found no support for the effects of temperature, predation, or their interaction on the total biovolume within dishes (Figure 5d, Table 2). However, we did find evidence for a positive relationship between species richness and total biovolume (Figure 5e, Table 2). For the biovolume ratio of predators to the remainder of the community, we again found no support for an effect of temperature relative to a null, intercept‐only model, although three of the communities at the intermediate temperature (24°C) displayed much higher biomass ratios compared to the other communities (Figure 5f, Table 2).

### 3.6 Respiration

Total community respiration displayed a unimodal relationship with temperature with a peak in respiration at intermediate temperatures and the lowest respiration at the highest and lowest temperatures (Figure 5c, Table 2).

## 4 DISCUSSION

Both predation and temperature can have substantial effects on the structure and function of communities, but we have little information about how predation and warming might interact to shape communities, even though we know that temperature alters interaction strengths (Englund et al., 2011; Gilbert et al., 2014; Rall et al., 2012; Uiterwaal & DeLong, 2020). This knowledge gap is critical, as the effects of warming will play out within communities of interacting species and changes in climate are in many places accompanied by predator extirpation or invasion. Our results indicate a systematic effect of temperature on community composition, while predator effects were detectable only at the intermediate temperatures at which the predator did well. Thus, it was difficult to identify a temperature–predation interaction per se, but it was clear that the predator had a narrower thermal niche than many of the species in the community, diminishing its impact on the system overall.

There are myriad predictions about changes in community structure caused by environmental warming, and these changes should be linked via the common underlying mechanisms driving demography and ultimately the abundances and occurrence of species within a community (Figure 1). For example, shrinking body size should simultaneously increase abundances and growth rates, while possibly also altering rates of predation and aggregate respiration. Similarly, if warming causes respiration to peak at intermediate temperatures for multiple species, then the aggregate ecosystem function should peak at intermediate temperatures and lead to higher biomass as well, regardless of species composition. We draw out some of the hypothesized links across levels of biological organization that may facilitate predictions of community‐level patterns (Figure 1). And while it is not yet completely clear how to make integrated predictions across individual to community levels of organization, the individual‐level phenotypes and interactions must lead in the aggregate to the population‐ and community‐level responses that we observe. Therefore, we would not go so far as to suggest our depiction of linked patterns (Figure 1) is a framework, but we do suggest that community‐level patterns should be linked in principle and therefore studied as a package along with individual‐level patterns.

The individual‐level property we measured in this study was body size. Many studies of species in isolation (Atkinson, 1994) and some field studies (Daufresne et al., 2009) suggest that individuals should grow to smaller size with warming (the temperature–size rule). Whether this shrinking emerges in communities, however, may depend on the full set of changes in resources, predation risk, and warming that individuals experience. In our microcosms, there was an about even split across species of individuals getting smaller with warming or the reverse, and some species either not responding in size or responding unimodally (Figure 2). This mix of responses makes it more challenging to offer simple predictions about climate change such as warming should increase abundances by lowering body sizes. The mixed size responses also would likely create a mixed set of changes in interspecific competition and predation susceptibility as well, leading to an overall shift in community structure perhaps like that we show in Figure 6. It could also influence the unimodal response of respiration to temperature, if on average individuals were smaller at high and low temperatures. This effect would likely augment the direct kinetic effect of temperature on respiration.

The mixed changes in size also may explain the idiosyncratic changes in species abundance distributions that we observed. Rank abundance curves (Figure 3) were steeper in predator dishes than in no‐predator dishes and varied across temperatures but not in a monotonic way. These shifts must somehow be linked to the changes in community structure (Figure 5), perhaps through changes in competition, apparent competition, or other types of interactions such as their own predator–prey interactions with bacteria. Even with such mixed species‐specific responses, intermediate temperatures still had higher overall metabolic function at intermediate temperatures (Figure 5f). It is not clear whether and how the increased metabolism at intermediate temperature played a role in facilitating or directing changes in community structure or population abundance in the presence of predators, or whether the higher metabolic rate at intermediate temperatures helped to fuel the productivity necessary to support the predators in the first place.

In our study, warming reduced species richness (Figure 5a). Such an outcome may have arisen from exceeding the thermal tolerances of a few species at the hotter temperatures. Indeed, 32°C exceeds the optimal growth temperature for at least some ciliates (Wieczynski et al., 2021), but shifts in interspecific competition also could have played a role (Bestion et al., 2018). This species richness change was positively related to a form of ecosystem functioning (biovolume) across microcosms. Such a biodiversity–ecosystem function (BEF) pattern has generally been observed through experimental manipulation of diversity (Tilman et al., 2014). But in natural systems, the interaction network and patterns of species responses to interactions and abiotic factors like temperature will determine how many species will persist in a system (Wieczynski et al., 2021). It is less clear that the BEF pattern should necessarily arise when diversity differs through self‐organization in a system than when it is set by manipulation. In our experiment, we saw a positive relationship between species richness and biomass across microcosms, indicating a BEF relationship. The variation in richness, however, was linked to temperature, with cooler treatments retaining more species. Warmer dishes, however, did not have higher biomass, so the origin of this pattern was warmer → less rich → lower biomass. This occurred despite the cooler dishes also having lower respiration.

Predator effects can be quite strong, but it is likely that many predator effects will be idiosyncratic, as they are likely to play out over some set of temperatures in which predators do well. *Actinosphaerium* was clearly limited to the intermediate temperatures in our experiment, being excluded in the warmest and coldest communities (Figure 4) and being the largest in the intermediate temperatures (Figure 2). The effect of predation, however, was not very consistent. Some predator dishes showed strongly divergent community composition and respiration than other dishes, indicating that for generalist predators like this one, there may be stochastic processes driving communities to different states depending on, perhaps, early patterns of predation that alter competitive interactions. This pattern might be different for more voracious predators, as *Actinosphaerium*, a sit‐and‐wait predator, could not eat all species in the community, and may have been itself predated by *Coleps*. The role of temperature in setting limits for predators could also arise through the prey. Two of the most frequent prey for the *Actinosphaerium* in this experiment were *Euplotes* and *Euchlanis* rotifers, both of which appeared to be the most abundant at intermediate temperatures.

To conclude, we advocate for more integrative observations of systems in response to climate change and predator losses or additions. Nonetheless, here we presented a mere “collection” of observations that are difficult to integrate, despite knowing that they must be connected through underlying mechanisms. Ecological science has long advocated for more integrative ways of understanding natural systems (McGill, 2010), but it remains difficult to implement. Even blackbox approaches to connect multiple measurements, such as structural equation modeling, would appear difficult to use in our case given the nonlinearities in our thermal responses and the few correlated measurements across scales. Nonetheless, our study does reveal some new insights. First, predator effects can be strong but relegated to relatively narrow thermal niches, implying that they could be lost from a system easily at high or low temperatures, as happened here. Second, temperature can reorganize a system while leaving some aggregate measures, such as species abundance distributions or total biovolume, relatively unchanged. Third, in contrast with many expectations stemming from metabolic theory (Brown et al., 2004), community respiration may decline at warmer temperatures, including those temperatures that are still “biologically relevant” for ecological function. And finally, the warming‐induced loss of species can reduce some aspect of ecosystem function. These results in combination differ in many respects from standing hypotheses, suggesting that finding ways to connect such patterns, perhaps through more mechanistic models, is sorely needed. Warming causes a wide range of physiological, morphological, and behavioral changes that may or may not lead to demographic changes, leading to changes in abundances, occurrence, and interactions. Although some generalizations in the dependence of patterns and processes on temperature are emerging, they currently need greater integration and generalization to more fully understand the ecological consequences of climate warming.

## AUTHOR CONTRIBUTIONS

**John P. DeLong:** Conceptualization (equal); data curation (equal); formal analysis (equal); methodology (equal); project administration (equal); writing – original draft (equal); writing – review and editing (equal). **Kyle E. Coblentz:** Data curation (equal); formal analysis (equal); investigation (equal); visualization (equal); writing – original draft (equal); writing – review and editing (equal). **Stella F. Uiterwaal:** Investigation (equal); writing – original draft (equal); writing – review and editing (equal). **Chika Akwani:** Conceptualization (equal); data curation (equal); investigation (equal). **Miranda E. Salsbery:** Investigation (equal); writing – original draft (equal); writing – review and editing (equal).

## FUNDING INFORMATION

This work was partially supported by NSF grant# 22‐513 Organismal Response to Climate Change (ORCC) to JPD.

## Supporting information


Data S1

## Data Availability

The data used in this paper along with code for analysis are available as Supporting Information.
