# Supplementary material for: Temperature and predators as interactive drivers of community properties
Source: Ecol Evol. 2023 Oct 31;13(11):e10665. doi: 10.1002/ece3.10665 (PMC10618570; doi:10.1002/ece3.10665)
Supplement: Supplementary file 1 — Data S1 [file ECE3-13-e10665-s001.zip › BiomassPyramidSuppMaterial.docx]

**Supplementary Material for**

**Temperature and predators as interactive drivers of community properties**

John P. DeLong^1^, Kyle E. Coblentz^1^, Stella F. Uiterwaal^1,2^, Chika Akwani^1^, and Miranda E. Salsbery^1,3^

**S1. Model Outputs for Body Size Responses**

**Table S1.1.** A table summarizing Generalized Additive Model (GAM) results for the top-performing models with biovolume of a species as the response variable. Only the top model is given except in cases where the null model and top non-null model were within 2ΔAICc scores. In these cases, the top non-model and null model results are given.

| ***Paramecium caudatum*** | | | | | | | | | |
| --- | --- | --- | --- | --- | --- | --- | --- | --- | --- |
| **Model** | **Coefficient/Smooth** | | **Estimate or Effective Degrees of Freedom** | | | | **t/F-value** | **p-value** | |
| **Temperature** | Intercept | | 1.7x10^6^ | | | | 16.91 | 2.2x10^-15^ | |
|  | Temperature (smooth) | | 3.465 | | | | 3.865 | 0.009 | |
| Deviance explained = 44.7% | | | | | | | | | |
| ***Paramecium aurelia*** | | | | | | | | | |
| **Model** | **Coefficient/Smooth** | | **Estimate or Effective Degrees of Freedom** | | | | **t/F-value** | **p-value** | |
| **Temperature** | Intercept | | 3.8x10^5^ | | | | 10.7 | 5.5x10^-11^ | |
|  | Temperature (smooth) | | 3.184 | | | | 3.537 | 0.027 | |
| Deviance explained = 37.9% | | | | | | | | | |
| **Euchlanis sp.** | | | | | | | | | |
| **Model** | **Coefficient/Smooth** | | **Estimate or Effective Degrees of Freedom** | | | | **t/F-value** | **p-value** | |
| **Temperature** | Intercept | | 2.8x10^5^ | | | | 17.64 | 3.27x10^-15^ | |
|  | Temperature (smooth) | | 2.1 | | | | 4.287 | 0.015 | |
| Deviance explained = 35.7% | | | | | | | | | |
| **Halteria sp.** | | | | | | | | | |
| **Model** | | **Coefficient/Smooth** | | **Estimate or Effective Degrees of Freedom** | | **t/F-value** | | | **p-value** |
| **Temperature** | | Intercept | | 2.1x10^4^ | | 42.62 | | | <2x10^-16^ |
|  |  | Temperature (smooth) | | 1 | | 3.474 | | | 0.075 |
| Deviance explained = 12.6% | | | | | | | | | |
| **Null** | | Intercept | | 2.1x10^4^ | | 42.55 | | | <2x10^-16^ |
| Deviance explained = 0% | | | | | | | | | |
| **Colpidium sp.** | | | | | | | | | |
| **Model** | | **Coefficient/Smooth** | | | **Estimate or Effective Degrees of Freedom** | | **t/F-value** | **p-value** | |
| **Temperature** | | Intercept | | | 1.4x10^4^ | | 18.71 | 1.5x10^-6^ | |
|  |  | Temperature (smooth) | | | 1 | | 7.425 | 0.034 | |
| Deviance explained = 55.3% | | | | | | | | | |
| **Null** | | Intercept | | | 1.3x10^4^ | | 13.91 | 2.34x10^-6^ | |
| Deviance explained = 0% | | | | | | | | | |
| **Frontonia sp.** | | | | | | | | | |
| **Model** | | **Coefficient/Smooth** | | | **Estimate or Effective Degrees of Freedom** | | **t/F-value** | **p-value** | |
| **Null** | | Intercept | | | 1.9x10^6^ | | 7.93 | 4.1x10^-7^ | |
| Deviance explained = 0% | | | | | | | | | |
| **Temperature** | | Intercept | | | 1.88x10^6^ | | 7.85 | 7.0x10^-7^ | |
|  |  | Temperature (smooth) | | | 1 | | 1.126 | 0.3 | |
| Deviance explained = 6.6% | | | | | | | | | |
| **Paramecium bursaria** | | | | | | | | | |
| **Model** | | **Coefficient/Smooth** | | | **Estimate or Effective Degrees of Freedom** | | **t/F-value** | **p-value** | |
| **Null** | | Intercept | | | 1.3x10^5^ | | 11.1 | 5.4x10^-10^ | |
| Deviance explained = 0% | | | | | | | | | |
| **Gastrotrich** | | | | | | | | | |
| **Model** | | **Coefficient/Smooth** | | | **Estimate or Effective Degrees of Freedom** | | **t/F-value** | **p-value** | |
| **Temperature** | | Intercept | | | 2.0x10^5^ | | 16.07 | 1.2x10^-13^ | |
|  |  | Temperature (smooth) | | | 1 | | 3.387 | 0.08 | |
| Deviance explained = 13.4% | | | | | | | | | |
| **Null** | | Intercept | | | 1.96x10^5^ | | 15.38 | 1.4x10^-13^ | |
| Deviance explained = 0% | | | | | | | | | |
| ***Coleps hirtus*** | | | | | | | | | |
| **Model** | | **Coefficient/Smooth** | | | **Estimate or Effective Degrees of Freedom** | | **t/F-value** | **p-value** | |
| **Temperature** | | Intercept | | | 4.3x10^4^ | | 27.63 | 3.4x10^-15^ | |
|  |  | Temperature (smooth) | | | 1.6 | | 16.48 | 0.0001 | |
| Deviance explained = 68.6% | | | | | | | | | |
| **Actinosphaerium sp.** | | | | | | | | | |
| **Model** | | **Coefficient/Smooth** | | | **Estimate or Effective Degrees of Freedom** | | **t/F-value** | **p-value** | |
| **Temperature** | | Intercept | | | 0.004 | | 8.14 | 8.7x10^-6^ | |
|  |  | Temperature (smooth) | | | 2.8 | | 4.46 | 0.025 | |
| Deviance explained = | | | | | | | | | |
| **Null** | | Intercept | | | 0.004 | | 17.31 | 5.9x10^-6^ | |
| Deviance explained = 0% | | | | | | | | | |

**S2 Model Outputs for Community-level Responses**

**TableS2.1** A table summarizing Generalized Additive Model (GAM) results for the top performing models with community-level variables as the response. Only the top performing model is listed unless the null model was within 2 ΔAICc units.

| **Temperature and Species Richness** | | | | | |
| --- | --- | --- | --- | --- | --- |
| **Model** | **Coefficient/Smooth** | | **Estimate or Effective Degrees of Freedom** | **t/F-value** | **p-value** |
| **Temperature** | Intercept | | 10.53 | 37.39 | <2x10^-16^ |
|  | Temperature (smooth) | | 1 | 12.95 | 0.0012 |
| Deviance Explained = 31.6% | | | | | |
| **Temperature and Shannon Diversity** | | | | | |
| **Model** | | **Coefficient/Smooth** | **Estimate or Effective Degrees of Freedom** | **t/F-value** | **p-value** |
| **Temperature** | | Intercept | 1.65 | 26.77 | <2x10^-16^ |
|  |  | Temperature (smooth) | 1.4 | 2.26 | 0.09 |
| Deviance explained = 16.3% | | | | | |
| **Null** | | Intercept | 1.65 | 25.1 | <2x10^-16^ |
| Deviance explained = 0% | | | | | |
| **Temperature and Respiration** | | | | | |
| **Model** | | **Coefficient/Smooth** | **Estimate or Effective Degrees of Freedom** | **t/F-value** | **p-value** |
| **Temperature** | | Intercept | 0.035 | 8.02 | 1.6x10^-8^ |
|  |  | Temperature (smooth) | 2.86 | 2.95 | 0.057 |
| Deviance explained = 31.8% | | | | | |
| **Temperature and Total Biovolume** | | | | | |
| **Model** | | **Coefficient/Smooth** | **Estimate or Effective Degrees of Freedom** | **t/F-value** | **p-value** |
| Null | | Intercept | 1.23x10^9^ | 12.24 | 5.56x10^-13^ |
| Deviance explained = 0% | | | | | |
| **Richness and Total Biovolume** | | | | | |
| **Model** | | **Coefficient/Smooth** | **Estimate or Effective Degrees of Freedom** | **t/F-value** | **p-value** |
| **Richness** | | Intercept | 1.23x10^9^ | 13.55 | 8x10^-14^ |
|  |  | Richness (smooth) | 1 | 7.39 | 0.011 |
| Deviance explained = 21.2% | | | | | |
| **Temperature and Biovolume Ratio** | | | | | |
| **Model** | | **Coefficient/Smooth** | **Estimate or Effective Degrees of Freedom** | **t/F-value** | **p-value** |
| Null | | Intercept | 0.11 | 2.05 | 0.057 |
| Deviance explained = 0% | | | | | |

**Table S2.2** A table summarizing linear model results for species abundance distributions with the natural log of relative frequencies of the response and abundance rank, temperature, and predator presence as factors interacting with abundance rank as the response. The intercept represents the abundance of a rank-0 prey, at 16 degrees Celsius, without predators.

| **Coefficient** | **Estimate** | **t-value** | **p-value** |
| --- | --- | --- | --- |
| Intercept | -0.42 | -2.52 | 0.012 |
| Rank | -0.48 | -21.24 | <2x10^-16^ |
| Temperature 20 | -0.38 | -1.81 | 0.07 |
| Temperature 24 | -0.17 | -0.76 | 0.45 |
| Temperature 28 | -0.83 | -3.80 | 0.00018 |
| Temperature 32 | -0.35 | -0.16 | 0.88 |
| Predator Present | 0.14 | 0.97 | 0.33 |
| Rank:Temp. 20 | 0.09 | 3.35 | 0.001 |
| Rank:Temp. 24 | 0.024 | 0.072 | 0.47 |
| Rank:Temp. 28 | 0.18 | 5.88 | 1.1x10^-8^ |
| Rank:Temp 32 | -0.096 | -2.75 | 0.006 |
| Rank:Predator Present | -0.078 | -3.74 | 0.0002 |
| Adjusted R^2^ = 0.87 | | | |
