# Supplementary material for: Temperature and predators as interactive drivers of community properties
Source: Ecol Evol. 2023 Oct 31;13(11):e10665. doi: 10.1002/ece3.10665 (PMC10618570; doi:10.1002/ece3.10665)
Supplement: Supplementary file 1 — Data S1 [file ECE3-13-e10665-s001.zip › Code Metadata.docx]

Biomass pyramid warming metadata for code

Community_Biovolume_Calculation.R – This R file uses the densities of prey from the ‘pyramid.csv’ file coupled with the sizes of organisms estimated using the FlowCam in ‘redoingcellsizedata.csv’ and the sizes of Actinosphaerium from ‘BP_Actin_2021.csv’ to calculate total community biovolume and the biovolume ratios of Actinosphaerium to the rest of the community. It then saves a new .csv file ‘Community_Data.csv’ containing these biovolumes along with information included in the original community data file ‘pyramid.csv’.

Pyramid_GAM_Analysis.R – This R file contains the code to perform the GAM analyses of the community-level covariates and uses AICc to compare the models. It also includes the code for the analysis of the rank abundance curves and for making a plot of Actinosphaerium densities.

NMDS_Analysis.R – This R file contains code to perform the NMDS and other analyses of community composition.
